# Policy, evidence and practice for post-birth care plans: a scoping review

**DOI:** 10.1186/s12884-019-2274-y

**Published:** 2019-04-25

**Authors:** Susan Crowther, Emma MacIver, Annie Lau

**Affiliations:** School of Nursing and Midwifery, Faculty of Health and Social Care, Robert Gordon University, Garthdee Road, Aberdeen, AB10 7QB UK

**Keywords:** Post-birth, Postnatal, Care plans, Continuity, Personalised care

## Abstract

**Background:**

Postnatal care continually attracts less attention than other parts of the childbirth year. Many regions consistently report poor maternal satisfaction with care in the post-birth period. Despite policy recommending post-birth planning be part of maternity services there remains a paucity of empirical evidence and reported experience using post-birth care plans. There is a need to report on post-birth care plans, identify policy and guideline recommendations and gaps in the current empirical research, as well as experiences creating and using post-birth care plans.

**Methods:**

This scoping review accessed empirical literature and government and professional documents from 2005 to present day to build a picture of current understanding of policy imperatives and existent published empirical evidence. The review was informed by the Arksey and O’Malley approach employing five stages.

**Results:**

The review revealed that post-birth care planning is promoted extensively in health policy and there is emergent evidence for its implementation. Yet there is a paucity of practice examples and only one evaluation in the UK. The review identified four overarching themes: ‘Positioning of post-birth care planning in policy; ‘Content and approach’; ‘Personalised care and relational continuity’; Feasibility and acceptability in practice’.

**Conclusions:**

Empirical evidence supports post-birth care planning, but evidence is limited leaving many unanswered questions. Health care policy reflects evidence and recommends implementation of post-birth care plans, however, there remains a paucity of information in relation to post-birth care planning experience and implementation in practice. Women need consistent information and advice and value personalised care. Models of care that facilitate these needs are focused on relational continuity and lead to greater satisfaction. It remains unclear if a combination of post-birth care planning and continuity of carer interventions would improve post-birth outcomes and satisfaction. Gaps in research knowledge and practice experience are identified and implications for practice and further research suggested.

## Introduction

The birth of a new baby heralds a time of transitions bringing new roles and responsibilities, demands and challenges for parents and families [1]. The quality of the postnatal support can have a significant impact on the post-birth experience and transition to parenthood. How care is delivered within this period greatly differs, depending on individual, organisational and cultural factors and maternal satisfaction is often reported as poor [1–5].

The concept of ‘postnatal’ is open to interpretation both in terminology and in its meaning. Officially defined by the World Health Organisation (WHO) as ‘ … *the period from childbirth to the 42nd day following delivery …* ’ [6], though, this is not universally accepted. Indeed, references elsewhere are made to this period extending up to 8 weeks [7]. Likewise, there are differences in what this period is named. References made in the literature and policy refer to ‘postnatal’, ‘postpartum’, ‘post-birth’ and the ‘fourth trimester’ [8] and are often used interchangeably. This article will use the term ‘post-birth’.

The face of post-birth care has changed markedly in the Western World in recent decades. The decrease in the length of stay in hospital is one of the most significant changes [9]. For example, in 1975, it was commonplace for women in UK hospitals to stay for at least 1 week after their baby’s birth, now 60% are home within 1 day of giving birth [10]. These shortened hospital stays are attributed to an increase in birth rates and a lack of resources in terms of midwives and hospital space [11]. Indeed, the Royal College of Midwives has estimated a current deficit of 3000 midwives in England [9]. Thus, for most women in the UK, post-birth care is provided in the home, initially by one or several different midwives visiting approximately three times [12], with responsibility passed onto health visitors from around 10 days after birth. Additional care, when required, is provided by other professionals such as General Practitioners and paediatricians.

Whilst some recent evidence suggests an improvement in levels of women’s satisfaction [4], generally, the quality and effectiveness of care provided in this period has been viewed by women and care providers in less positive terms than antenatal and perinatal care. This finding is not unique to a UK context, but extends to Australia, the U.S. and Belgium too [1–5, 13–20]. This has led to the notion of post-birth care being viewed as the ‘poor relation’ or the ‘Cinderella’ of services [21].

Addressing satisfaction is multifactorial and proves challenging. Women often report unmet needs for information, guidance and support on a range of issues including common post-birth health concerns and self-care [3, 22] post-natal depression [3]; baby-care and support with breastfeeding [3, 23]; and not having an opportunity for a birth de-brief [3]. Additionally, given that care in the post-birth period is frequently provided by several different professionals, fragmented care, poor communication and lack of individualised information has become a common complaint [8].

This paints a picture of an under-resourced, under-valued and poorly managed part of the maternity service which is particularly worrying given that women and infants face increased vulnerabilities in the post-birth period compared to any other period in their antenatal or perinatal journey [8, 24, 25]. Consequently, the provision of appropriate post-birth care plays a vital role in addressing potential vulnerabilities [26].

In response to these concerns policy has broadly called for a postnatal service that responds to women’s physical, psychological, emotional and social needs [27]. Policy drivers expect care to be personalised and individualised with a focus on continuity and integration of care [28, 29], underpinned by the principles of kindness, dignity, respect [30] and informed choice [29]. Indeed, a recent Scottish policy review has highlighted the following four areas as being of importance to women: continuity of care and carer; more information and choice; better emotional support and better access to services locally [29].

In terms of care provision, women need to be fully involved in the planning and timing of postnatal visits [30]. To fully embrace this, NICE introduced the notion that all postnatal women should have an individualised post-birth care plan (PBCP) discussed and developed in partnership with women in the antenatal period [7]. Despite this, and the reiteration of this message in their updated 2015 guidelines, and an array of other policy documents both in the UK and across other countries, there continues to be minimal evidence of PBCP being used within practice [2]. Table 1 outlines the main thrust of relevant policy and recommendations in relation to the UK, US and Australia where guidelines like the NICE guidance on PBCP are evident. These are presented in chronological order to provide an understanding of policy development over time.

**Table 1:** A Chronological Account of Post-Birth Care Plans in Policy

Policy document, Author and Year of Publication	Country	Overview and Relevance to Post-Birth Care Planning
National Service Framework (NSF) for Children, Young People and Maternity Services Department of Health and Social Care 2004	England	Emphasises the importance of a fully personalised care plan spanning pregnancy, childbirth and the post-birth period, but lacks detail on what this should encompass postnatally. States the importance of *‘continuity of support’* throughout the maternity journey as well as an *‘individualised, flexible, woman-focused approach to care and support’*.
National Institute for Health and Care Excellence (NICE), − Postnatal care up to 8 weeks after birth CG 37 NICE 2006 (last updated in 2015)	UK	Introduced the idea of post-birth care plans, stating that *“a documented, individualised postnatal care plan should be developed with the woman ideally in the antenatal period or as soon as possible after birth”* (1.1.3). These need to be tailored to meet the needs of each woman and include relevant factors from the antenatal, intrapartum and post-birth period and revisited at each contact. It is stated that a well-developed plan would improve continuity of care.
Maternity Matters Department of Health 2007	England	‘Personalised care plans’ for the antenatal period and birth are mentioned, no specific mention of extending this to the post-birth period. Highlights the importance of both personalised care and continuity of the care-giver throughout pregnancy and into the post-birth period.
Pathways for Maternity Care NHS Trust March 2009	Scotland	Reiterates the importance of an individualised care plan as per NICE guidance Professional support should be individualised according to the needs of the woman and baby. Continuity of care/ carer should be encouraged both antenatally and postnatally.
A Refreshed Framework for Maternity Care in Scotland The Maternity Services Action Group Scottish Government 2011	Scotland	No explicit reference to PBCP, but states that post-birth care should be delivered in line with national guidelines (including the NICE guidelines). Also, makes a brief reference to ‘maternity care planning’, but does not elaborate on what this entails. Women and babies should have an assessment of their needs with ongoing assessment at every post-birth contact. Recognises the importance of personalised care and continuity of care and carer, but does not explicitly state that this should be the same midwife for antenatal and post-birth periods.
Postnatal Care Program Guidelines for Victorian Health Services, State of Victoria’s Department of Health and Human Services 2012	Australia	Recommends that post-birth care planning starts during the antenatal period and should include the woman’s preferred location and timing of her care. Post-birth care should be “women-centred”. Promotes continuity of care and carer throughout the maternity care pathway.
Optimizing Postnatal Care American College of Obstetricians and Gynecologists (ACOG) 2016	USA	Planning for post-birth care should begin during pregnancy by developing a postpartum care plan specific to each woman. Continuity of care and good communication are key. Post-birth care planning should be based on discussions intimating a conversational style.
National Maternity Review - Better Births NHS 2016	England	Recommends that all women have a ‘personalised care plan’ for their whole maternity pathway. Highlights the importance of more personalised care. Post-birth care should be led by the woman’s named midwife who should assist the woman in developing the post-birth part of her personalised care plan.
The Best Start: A Five-Year Forward Plan for Maternity and Neonatal Care in Scotland - Executive Summary Report Scottish Government 2017	Scotland	Options for post-birth support should be discussed by woman and midwife during pregnancy and the woman’s decisions recorded in a shared personalised care plan, reviewed throughout the maternity journey. Provides key recommendations around the ‘continuity of carer’, an individualised model of care, and keeping woman and baby at the centre of care.
Implementing Better Births – a Resource Pack for Local Maternity Services NHS 2017	England	All women should have a personalised care plan for the whole maternity journey. The post-birth part of the plan should be considered before the birth and revisited throughout. All women should be offered assistance and support to form the care plan but it should be ‘owned’ by the woman. The discussion that informs the care plan should be viewed as a ‘conversation’.

At the time of this review a funded project is underway in NE Scotland examining the creation, acceptability and feasibility of a PBCP to help address the above needs and vulnerabilities with the hope of improving post-birth care, maternal satisfaction and address current policy recommendations. In the initial stages of this project it was apparent that a scoping review of current policy and empirical evidence in this domain was needed to collate and synthesise what was happening in this domain and provide a comprehensive presentation of the current knowledge and practice.

### Search strategy

A systematic scoping literature review was adopted because this allowed for a broad sweep of the available policy and evidence to identify salient themes and highlight key concepts of the domain. Arksey and O’Malley (2005) five stage approach ensured a systematic process was followed [31].

### Stage one: identifying the review question

This was developed through the usage of a PICO analysis (see Table 2). Critically, the research question reflected a concurrent primary study exploring the development and feasibility of a PBCP in a Scottish maternity service.

**Table 2:** PICO analysis for development of the research question

	PICO Headings	Description of Areas Included
P	Participants	Pregnant women (and their partners/ families), midwives
I	Intervention	PBCP
C	Context	Middle-to high income countries, English-speaking; countries with a maternity-care infrastructure, 2006 – present day to reflect the initial guidelines issued by NICE regarding the need for post-birth care planning
O	Outcome	Women’s satisfaction with their post-birth care. Women being involved in decisions about their own care and their baby’s care. Provides an opportunity for women to identify and predict their own post-birth care needs

The question for this review was subsequently *‘What is known from the existing literature about women’s and midwives’ experiences, views and perspectives of post-birth care plans?’*

### Stage two: identifying relevant studies/articles/papers

This involved identifying relevant studies to address the stated review question. A comprehensive search was developed. A range of databases were identified as relevant: CINAHL, Intermid, Cochrane Library, Scopus, Science Direct, OVID, MIDIRS. The basic search terms used were: “post-birth” OR post-birth OR postpartum OR “post partum” OR post-partum OR postnatal OR post-natal OR “post-delivery” OR “post delivery” AND “care plan*” OR document OR initiative OR plan* OR template OR strategy* OR framework OR structure OR arrangement OR design OR support. Critically, this search set out to encompass all forms of the term ‘postnatal’ and a comprehensive range of terms meaning or indicating ‘care plan’.

Some of the databases required the terms to be imputed in a slightly different format, but ultimately the same search terms were applied. The search terms evolved over time to reflect growing understanding and awareness of the area of interest. Where this occurred, searches were repeated with the inclusion of new search terms. For instance, the earliest searches did not include the terms ‘strategy’, ‘template’, ‘framework’, ‘structure’, ‘arrangement’, ‘design’. Boolean operators and wildcards were applied as necessary to ensure the most thorough and comprehensive search. Also, the initial searches uncovered material relating to areas outside the area of interest, such as ‘family planning’ and ‘postnatal depression’. Thus, later modified searches excluded these.

**Fig. 1:**
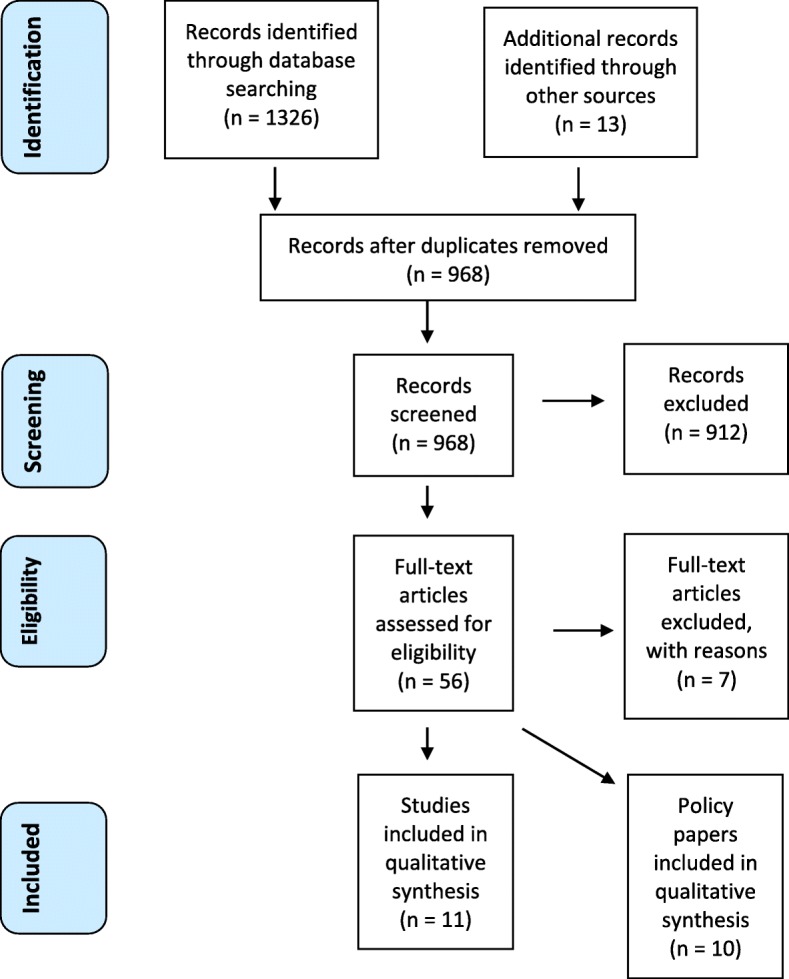
PRISMA Flow Diagram

In addition, search engines Google and Google Scholar and relevant websites, including the National Health Service (NHS), National Childbirth Trust (NCT), The Royal College of Midwives (RCM) and National Institute for Health and Care Excellence (NICE) were explored. The search was performed between July and September 2018 and a further search in November 2018 to identify subsequently published articles. Studies were included if published between 2005 and 2018 – the chosen starting point reflected the time immediately preceding the publication of critical policy in the area, the NICE guidelines on postnatal care plans [7]. A chronological record of the entire search is provided in the PRISMA chart in Fig. 1.

### Stage three: study/article/paper selection

Abstracts of all publications identified were checked for relevance to the current review. Studies and papers were included if they met the following criteria:

- Published between 2005 until November 2018.
- Focus primarily on women’s and midwives’ views on PBCP.
- The language limited to English only (due to limited time and budget constraints – specifically, the cost of translation).
- Papers and studies from Organisation for Economic Co-operation and Development (OECD) countries with established systems of maternity care.
- Focus explicitly on post-birth care planning and not specific post-birth morbidities and care needs.

It is important to highlight that whilst the focus of the current review was concerned with English-speaking, middle to higher income countries with an established maternity care infrastructure all potential sources were examined. Although there was an inclusion criterion of English language an additional search was done to include non-English papers applying the original search terms to ensure that no other seminal or significant knowledge in this area was omitted. Articles in Finnish, Chinese, Japanese, Persian and Spanish were found, and their English titles and abstracts screened, however, none of these were relevant to PBCP or the aim of this review and were therefore excluded. Our search did include one paper published in English, relating to the Belgium-context. All relevant materials were available in full-text forms and all of these were accessed and if relevant non-English papers had been identified these would have been translated and included. Secondary searchers of the reference lists of selected articles were undertaken to check for relevant publications to ensure that the search was as comprehensive as possible. Several sources were excluded at this stage if inclusion criteria were not apparent.

### Stage four: charting the data

The fourth stage in the process involved recording key data, synthesising and interpreting the qualitative information according to key themes. Arksey and O’Malley (2005) suggest that key data in the review be presented in tabular format, which in this case involved charting the key information and data relating to relevant policies and research papers [2, 31]. This includes the author, year of publication, study location, population, aims, methodology and findings. Table 1 presents an overview of relevant policy, whilst Table 3 outlines relevant evidence concerning the use of PBCP in practice.

**Table 3:** Evidence Concerning Use of Post-Birth Care Plans

Document, Author and Year	Location	Method and Participants	Main aims/ objectives/purpose	Main findings
Left to your own devices: The postnatal care experiences of 1260 first-time Mothers Newburn and Bhavnani, (NCT) 2010	UK	Findings of a survey carried out with 1260 first-time mothers - largely NCT members - who had given birth to their first baby during 2008–2009.	To investigate the post-birth experiences of women, the quality of support in the first few weeks after giving birth. Considered the extent to which the NICE recommendations on the use of individualised post-birth care plans had been implemented.	96% of women stated that they had not been not involved in developing a post-birth care plan as recommended by NICE. Many women reported poor co-ordination and planning of care they received and a lack of continuity of carer.
Pressure Points Campaign – ‘Postnatal Care Planning’ Royal College of Midwives (RCM) 2014	UK	In 2013, the RCM surveyed midwives, maternity support workers and student midwife members from across the UK. They also asked women for their experiences of post-birth care.	To investigate the extent to which post-birth care plans are used, barriers to use and experiences, from the perspective of women and professionals.	There’s a significant gap between what women should be receiving in terms of post-birth care planning and what they are getting. Almost half of the women could not recall discussing a post-birth care plan before the birth and 2/3 had not discussed it afterwards. A lack of resources and lack of professional awareness of the NICE guidance was identified as the main reasons for this. Reiterates the importance of continuity of care and individualised care.
Safely delivered – a national survey of women’s experience of maternity care 2014 Redshaw, M. & Henderson, J. National Perinatal Epidemiology Unit (NPEU) 2014	England	Based on a survey of 4571 women, who gave birth during a 2 week period in 2014.	Set out to investigate women’s experiences of their maternity care, including post-birth care.	The participants were not asked whether they had completed a post-birth care plan. Consideration of individualised care and continuity of carer. Whilst most women were satisfied with their care received at all stages, lesser levels of satisfaction were evident in relation to post-birth care.
Individualised, flexible postnatal care: a feasibility study for a randomised controlled trial Forster, D. et al. 2014	Australia	109 women approached during pregnancy, were sent a postal survey 8 weeks after giving birth, completed by 67. Clinical data was collected from medical records.	The study refers to ‘planning in the antenatal period for postnatal care’ and a ‘postnatal care plan’, but the investigation is concerned solely with a new proposed model relating to early hospital discharge.	Most women (*n* = 103) completed a ‘postnatal care plan’ during pregnancy; 17% planned to leave hospital within 12 h after birth and 36% planned to stay 48 h. The ‘postnatal care plan’ considers the family support system at home.
Having a baby in Scotland 2015: listening to mothers An Official Statistics publication for Scotland Scottish Government 2015	Scotland	Survey of 2000 women who gave birth during February and March 2015.	The aim of the study was to provide a benchmark for improvement in maternity services and inform a major review of maternity services in Scotland. Very similar to the CQC survey in England.	The survey failed to directly ask women whether they had completed a post-birth care plan. Most women reported a very positive experience of their maternity care. Post-birth care was viewed as less positive than antenatal and intrapartum care. Reiterates the importance of continuity of care and carer and the provision of individualised care.
A Survey of Women’s Experiences of Maternity Care in Northern Ireland National Perinatal Epidemiology Unit (NPEU) and the School of Nursing and Midwifery at Queen’s University 2016	Northern Ireland	Details the experiences of 2722 women who gave birth between October 2014 and December 2016.	Set out to uncover women’s experiences of maternity care in Northern Ireland.	There was no question posed around post-birth care plans or broader maternity care planning. Similar findings to comparative surveys in England and Scotland, though generally the women were more satisfied with their post-birth care than the other countries. Recognition of the challenges regarding continuity of care. The importance of providing individualised care is recognised as central.
Support Overdue: women’s experiences of maternity services National Childbirth Trust (NCT) and National Federation of Women’s Institutes (NFWI) 2017	UK	Survey of 2500 women who gave birth in England or Wales in 2014, 2015, and 2016.	The study set out to investigate women’s experiences of maternity services.	States that the NCT endorse the NICE guidelines around developing a post-birth care plan, yet there is no specific question asking whether the women had developed a plan or what it contained. The report outlines some key areas, including continuity of carer, and personalisation – though the latter is discussed mainly in relation to antenatal care.
A systematic approach towards the development of quality indicators for postnatal care after discharge in Flanders, Belgium Helsloot, et al. 2017	Belguim	Involved health care professionals, representatives of health care organisations and policy makers with expertise in the field of post-birth care.	Set out to develop a set of quality indicators for post-birth care after hospital discharge, to monitor and improve care provision.	Identified a range of ‘quality indicators’. States that planning for post-birth care should start in pregnancy with an *individualised care plan* that follows mother and baby throughout the pregnancy, birth and post-birth periods. There is no template of such a plan offered. Some recognition around the ‘feasibility’ and ‘acceptability’ of such a care plan, but limited elaboration of this. The importance of continuity of care is emphasised.
Your Birth We Care – a survey exploring women’s experiences in pregnancy and birth in Wales Welsh Government 2017	Wales	The survey was completed by 3968 mothers from all over Wales.	It aimed to understand the perception of women about the quality of antenatal care and the capacity of the service to prepare women for labour, birth and also parenting.	As this survey was primarily concerned with pregnancy and birth, there was limited mention of post-birth care and needs and no discussion of care planning for either the post-birth period or any other part of the maternity journey. Emphasised the importance of continuity of care and carer and the challenges in achieving this. Individualised care was emphasised, but only in terms of antenatal care and birth choices.
Survey of women’s experiences of maternity care Care Quality Commission 2018	England	Large-scale maternity survey based on responses from 18,426 women who gave birth during January–February 2017.	It aimed to uncover women’s experiences of their care during labour and birth, as well as the quality of antenatal and post-birth support.	Refers to the NICE guidelines on developing a post-birth care plan, but does not ask women whether they had had the opportunity to develop a plan. Compared to previous surveys, the largest improvements were in post-birth care - though this was still viewed less positively than other aspects of maternity care. The importance or continuity of care and individualised care is recognised.
Planning for your Postnatal Care Needs Personal Care Plans – for mums and families North-West London Sustainability and Transformation Plans (STP) 2018	England	An initial consultation with women from the North-West London area. Development of a postnatal care plan tool in line with the NICE guidelines. Subsequent pilot phase and evaluation of this tool by 27 women and 4 midwives via a survey and several others in small focus groups, resulting in the reworking of the tool and development of a maternity care planner.	Setout to develop ways in which local post-birth care service provision could be improved, in terms of information needs, and promoting continuity of care and personalised care. The pilot sought to address: the feasibility of using the too; its usefulness in signposting women to relevant information; and its effectiveness in preparing women for parenthood.	In terms of the findings: the majority of the women felt that the plan: had been introduced at the right time; had helped to prepare for post-birth needs, requires to be addressed through face-to-face conversations with their midwife. The midwife feedback was generally positive. The tool was viewed as helpful in planning for post-birth care and particularly for first-time mothers, but seen as creating additional time pressures and training needs.

## Findings

### Stage five: collating, synthesising and reporting findings

The fifth stage involved collating, summarising and reporting the findings. There were a range of methodologies, types of data and a variety of sources. This included qualitative studies, mainly employing survey methods, as well as policy papers and guidance. The thematised findings are a result of synthesis of the included data and information. The findings are organised and presented in four overarching themes: ‘positioning of post-birth care planning in policy; ‘content and approach’; ‘personalised care and relational continuity’; ‘feasibility and acceptability in practice’. These themes are highlighted across the various papers, as summarised in Table 4, and reported individually.

**Table 4:** Broad Themes Across the Various Policy and Research Papers

Publications	Positioning of PBCP in policy	Content and approach	Personalised care and relational continuity	Feasibility and acceptability in practice
National Service Framework (NSF) for Children, Young People and Maternity Services (2004, UK)	√		√	
National Institute for Health and Care Excellence (NICE), NICE Clinical Guidelines, No 37 (2006 - last updated in 2015, UK)	√	√	√	
Maternity Matters, Department of Health (2007, England)			√	
Pathways for Maternity Care NHS Trust (2009, Scotland)		√	√	
Newburn and Bhavnani, (NCT) Left to your own devices: The Postnatal Care Experiences of 1260 first-time Mothers (2010, UK)		√	√	
A Refreshed Framework for Maternity Care in Scotland (The Scottish Government) (2011, Scotland)	√	√	√	
Postnatal Care Program Guidelines for Victorian Health Services, from State of Victoria’s Department of Health and Human Services (2012, Australia)		√	√	
Royal College of Midwives (RCM), Pressure Points Campaign – Postnatal Care Planning (2014, UK)		√	√	√
Redshaw, M. & Henderson, J. (NPEU) Safely Delivered – a national survey of women’s experience of maternity care (2014, England)			√	
Forster, D. et al, Individualised, flexible postnatal care: a feasibility study for a randomised controlled trial (2014, Australia)		√	√	√
The Scottish Government, Having a baby in Scotland 2015: listening to mothers (Scotland, 2015)			√	
American College of Obstetricians and Gynaecologists (ACOG) (2016, USA)		√	√	
NPEU and Queen’s University, A Survey of Women’s Experiences of Maternity Care in Northern Ireland (2016, Northern Ireland)			√	
National Maternity Review - Better Births (2016, England)	√	√	√	
Implementing Better Births – a Resource Pack for Local Maternity Services (2017, England)	√	√	√	
National Childbirth Trust (NCT) and National Federation of Women’s Institutes (NFWI), Support Overdue: women’s experiences of maternity services (2017, UK)	√	√	√	
Welsh Government, Your Birth We Care – a survey exploring women’s experiences in pregnancy and birth in Wales (2017, Wales)			√	
The Best Start: A Five-Year Forward Plan for Maternity and Neonatal Care in Scotland - Executive Summary Report, The Scottish Government (2017, Scotland)	√	√	√	
Helsloot, et al. A systematic approach towards the development of quality indicators for postnatal care after discharge in Flanders, Belgium (2017, Belgium)	√		√	√
Care Quality Commission, Survey of women’s experiences of maternity care (2018, England)	√		√	
North-West London STP, Personalised Post-Natal Care Plan, Evaluation and Personal Care Plans for Mums and Families (2018, England)	√	√	√	√

### Positioning of post-birth care planning in policy

NICE (2006) for the first time in policy called for all women to have the opportunity to develop a documented and individualised PBCP [7]. It was recognised that antenatal and intrapartum factors should be considered when devising this plan, and states clearly ‘Postnatal care should be a continuation of the care’ [7]. These recommendations are reiterated across a range of later policy documents [32, 33], with similar guidance evident in other developed countries [34, 35]. Nevertheless, it is not explicitly stated that a PBCP should be included as part of a wider maternity care planning tool in either the original NICE recommendations or the later updates. Nevertheless, various Scottish and English policies [4, 29, 36, 37], call for maternity care planning that encompasses all stages of the maternity journey, arguably offering a more holistic approach in the consideration of the needs of women and families. This would be based on the woman’s decisions and wishes around her pregnancy, plans for giving birth and her post-birth needs. These needs and wishes may include anything of importance to the woman, but should include the woman’s values, expectations, fears and concerns around her pregnancy and her new role as a mother, as well as exploration of her support networks. These objectives are emphasised across various papers [38, 39].

### Practice examples

Despite these calls for either a PBCP as a standalone tool or as part of a wider maternity care planner, policy-makers have failed to offer any template or working model for service-providers and women to utilise. Evidence suggests that these plans are not being used in practice [1, 2]. Moreover, the only example available in the public domain started as a PBCP, but following evaluation, was reworked and later included as an element within a wider maternity planning tool [40]. Perhaps this merits discussion of whether firstly, the PBCP be considered as a standalone tool or be incorporated into a more comprehensive maternity care plan and secondly, what this should ‘look’ like. Moreover, this tool was based on a project relating to one specific geographical location in the UK [40] and thus, it is unclear whether their experience could be transferable to other settings that have their own unique features and characteristics.

Despite the clear expectation of post-birth care planning set-out in policy, there is little evidence of such care plans being utilised in practice. Much of the literature and policy documents quantify women’s experience of postnatal services, that is, describe the number and timing of post-birth visits, rather than discuss the content of visits [41]. Adequate contact from professionals is important in ensuring that women receive appropriate care during the post-birth period [6, 7]. Yet, Kearns et al. contend that more emphasis be placed on how post-birth contact and support addresses the needs of women and that the number of contacts alone with the health system has limited value [42]. They also suggest that this issue reflects a lack of guidance for professionals around what should be covered within post-birth visits and planning [42]. Indeed, as Morrow et al. highlight there is a paucity of studies that focus on implementation and evaluation of post-birth interventions aimed at improving care [43].

An extensive search revealed very few examples of working PBCP, and research evidence reiterates limited use of these in practice and only one evaluated tool [40]. The search was based on a consultation with women from North-West London around their post-birth care needs, which revealed issues around inconsistent and confusing advice from professionals and a need for continuity of care and personalised care [5, 40]. Based on their review findings, the researchers set about developing ways in which local post-birth care service provision could be improved.

As well as publishing an information booklet to improve the quality, quantity and accuracy of information provided to women and families [40], the NICE recommendation around postnatal care planning was addressed. Subsequently a PBCP was developed by a multidisciplinary team of professionals providing maternity services along with service users and piloted by a community team from each Trust in the North-West London area. This PBCP was to be introduced by the named midwife in the antenatal phase at 36–38 week and thereafter, revisited and discussed by women and their midwives during every subsequent appointment in the antenatal and post-birth periods. In line with the NICE guidelines, the plan included relevant information from the woman’s notes regarding her antenatal, intrapartum and post-birth stages, as well as contact details of key health professionals. The expected benefits of utilising this tool in practice were stated to include: “better care information and clinical outcomes for women and babies, improved experience for women and their families, and better communication between women and healthcare professionals” [40].

In the pilot phase, 250 plans were distributed, and the tool was thereafter evaluated by 27 women and 4 midwives via a survey and several others in small focus groups. The evaluation sought to address the feasibility of using the tool in the way prescribed, its usefulness in signposting women to relevant information and services and the effectiveness of the tool in preparing women for parenthood and caring for a baby. They found that most of the women felt the plan had been introduced at the right time antenatally and around half, had had the opportunity to discuss the plan on multiple occasions in the antenatal and post-birth periods. Most of the women agreed that the plan had helped them to prepare for their post-birth needs, particularly in terms of their own and their baby’s health and wellbeing. Further important findings were around women’s information needs, the clarity and design of the tool, the relevance of the topics in the tool and the importance of exploring emotional wellbeing. Most women felt that the information provided using the tool was helpful but additional information on practical as well as self-care and baby care issues (e.g. room temperature, using the toilet after birth, feeding) was required. Women also reported that questions/ areas discussed should be open and form the basis of a discussion, rather than closed yes/ no type question. Many of the women in the evaluation preferred to discuss their post-birth care needs antenatally, although some women found this overwhelming and wanted to wait until after their baby’s birth. Overall the women reported the importance of talking about emotional wellbeing and mental health and how to access help if required and that face-to-face conversations about post-birth needs, rather than solely written information, is preferred.

In terms of the midwives’ feedback, it was acknowledged that the tool was helpful in planning for post-birth care and particularly useful for first-time mothers as well as partners/ fathers. Nevertheless, it was recognised that it may create additional time pressures if women wish to discuss many topics, it may not be as useful for second and subsequent pregnancies and training on how to use the tool would be essential. Reflecting the women’s comments, the midwives felt that open space to add comments in the plan would be useful, intimating the need for more open discussion. Lastly, the midwives felt that the form would be most useful if presented in a digital format. Most of these issues were addressed, and the tool was reworked and expanded to include all of the parts of the care pathway (antenatal, intrapartum and post-birth) in line with the recommendations set-out in ‘Better Births’ [37].

A further two post-birth plans found during the literature search were developed by Doulas for the use of women in their care, though these are widely available for other women to access online (e.g. sarahtessier.com). These contain specific areas for consideration, mainly practical issues, self-care, baby care, emotional support as well as the woman’s support network. Nevertheless, these have not been evaluated and are presented as ‘standalone’ documents with little explanation of their background, use or development.

### Content and approach

Whilst there is only one available example of an evaluated post-birth plan, there is some guidance provided in policy documents around the areas that should be included in a PBCP or maternity care planning tool [7, 32–35, 37, 38, 40]. The content and approach of post-birth care planning is considered here in four questions: ‘what’, ‘who’ ‘when’ and ‘how’.

### What information?

According to NICE [7] and echoed in the American guidelines [35], the PBCP should consider relevant issues from the antenatal, intrapartum and postnatal periods and include areas such as: the woman’s adjustment to motherhood; physical health needs of the woman and baby; emotional wellbeing and recognition of mental illness; available family support; feeding plans including support with preferred method (breast or formula feeding). These policies, as well as the Victorian guidelines, also state that the details of the healthcare professionals involved in the care of woman and baby (including their roles and contact details), should be clearly stipulated [7, 34, 35]. Other policy papers in the UK reassert these areas [37, 38] and several research papers recommend that the NICE guidelines are adhered to [1, 2, 44]. Moreover, these areas are reflected in the postnatal section in the maternity care plan already discussed, organised under the broad headings: physical and emotional wellbeing; caring for baby; immediate post-birth issues; practical issues and birth reflections [40].

Some of these areas are elaborated on in other policy documents. For instance, ‘physical health’ may encompass the effects of nicotine and substance use on mother and baby, maternal and infant nutrition, oral health and the prevention of accidents in the home, contraception and family planning [33], as well as the impact of and planning around ‘existing medical conditions’ or medical issues in the family [38]. Additional considerations, such as caring responsibilities, employment and safeguarding risks are highlighted elsewhere [38].

In planning for women’s individualised post-birth care, there is a very apparent need for good quality, relevant and timely information and advice to enable women to make informed choices. This is recognised within various policies [7, 38] and was put into practice by the North-West London STP, in their provision of an information tool to accompany the maternity care planner [40]. There is also recognition that the woman’s choice around her preferred location of her post-birth care requires consideration in this planning [2, 4, 34, 45].

### Who develops and delivers?

The question of ‘who’ relates to firstly, those involved in developing the care plan and secondly, those delivering the care in the post-birth period. It is generally viewed that women and midwives are the key players in devising the plan and midwives are central to post-birth care delivery, with assistance from and handover to other relevant health professionals as appropriate, including health visitors and General Practitioners [37]. This has been elaborated on in some policy documents, though the emphasis on the role and responsibilities of the key players varies. For instance, in the English context, it is recommended that women should be ‘assisted’ to develop the post-birth element of the maternity care plan, with the process being ‘led’ by the named midwife [37]. The Victorian guidelines, however, describe this process as being ‘women-centred’, positioning the woman as ‘key driver’ in planning her post-birth care [34].

### When should post-birth care planning occur?

‘When’ relates to firstly, the point at which post-birth care planning should be undertaken and secondly, when post-birth care should be provided. Both the NICE [7] and Victorian guidelines [34] recommend that post-birth care planning should ideally start during the antenatal period (or if not possible, very shortly after birth) and should include the woman’s preferences around the timing of post-birth care provision - though it’s stressed elsewhere that the number of post-birth contacts should not be pre-determined [2]. The plan should be revisited at each postnatal contact [7] to reflect evolving circumstances and choices.

### How should a PBCP be used?

The approach in post-birth care planning is moving away from didactic teaching to a dialogue which honours the woman’s needs within her contexts. The evidence from the maternity care policy echo with the change in emphasis on the woman and a conversational focus. The ACOG recommends that planning for post-birth care should be ‘discussed’ indicating a conversational style between the woman and the maternity care team [35]. The Best Start report [29] states that options for post-birth support should be ‘discussed’ by woman and midwife during pregnancy and the woman’s decisions recorded in a ‘shared plan’ along with key decisions about her pregnancy and birth, again inferring a more conversational approach.

A personalised care plan should acknowledge unique individual circumstances and be reviewed jointly between the woman and her midwife throughout the maternity journey. Implementing Better Births reiterates that the personalised maternity care plan should be ‘owned’ by the woman, rather than the professional, shared with other care providers as necessary [38]. Again, it is stated that the discussion informing the care plan should be viewed as a ‘conversation’ between the woman and her midwife. Moreover, whilst the National Maternity Review does not explicitly refer to discussions or a conversational approach with the woman, it is recognised that choice, involvement and more personalised care are key and, that care planning should be reviewed jointly throughout the maternity journey [37].

### Personalised care and relational continuity

Evident in the previous theme on content and approach is the need for post-birth planning to be individualised and personalised. Personalised care is key to providing important information at the appropriate time, recognising when support is needed and offering targeted interventions through the appropriate multi-agency care pathways [2]. Confusingly, the terms personalised and individualised are used interchangeably in the literature. We found 32 instances of the term ‘personalised’ and 27 instances of the term ‘individualised’ in the reviewed literature. For the purposes of this review we continue with using the term personalised to mean both terms.

Although both the Victorian guidelines on post-birth care [34] and NICE guidelines on the routine care of postnatal women and their babies [7] suggest all care should be individualised there is a lack of evidence regarding the efficacy of a personalised approach to post-birth care and whether this would be feasible from an organisational perspective [11]. However, contemporary policy continually reiterates that all women should have a personalised care plan for the whole maternity journey [38]. Personalised care is care centred on the woman, her baby and her family, based around their needs and their decisions; where women are informed by unbiased information to make informed choices about the place of birth, pain relief and practical help and assistance with personal care, as well as post-birth care needs and wishes. A promising way to facilitate this personalised approach is through models of care that enable relational continuity.

Evidence clearly identifies relational continuity as being at the heart of best evidence-based midwifery practice consistently affording improved clinical and psychosocial outcomes for women and their babies [45]. Continuity of carer is a model of care that enables the opportunity for relational continuity and is continually recommended in national UK guidance [7]. Likewise, the ACOG recognises that continuity of care and good communication amongst healthcare providers in planning post-birth care is crucial for optimal outcomes [35]. The significance of relational continuity as enabling personalised post-birth care is a broad theme that extended throughout all 11 studies and 10 policy papers related to post-birth care planning and presented here through four themes: Acceptability and desirability, Trust and compassion, Supports decision-making and Supports post-birth care planning.

### Acceptability and desirability

Evidence for continuity of carer as acceptable and desirable is growing [2, 46]. The RCM report that relational continuity is a very significant factor in determining the satisfaction women feel when reviewing their post-birth care [2]. The preference for relational continuity is strongly representative across many surveys and consultations with maternity service users [14, 37, 44]. The importance of building relationships with the same person or team throughout care is important to women [29] and the ‘Maternity Matters’ document [47] stressed the importance of personalised care and continuity of the care-giver throughout pregnancy and into the post-birth period. Half of the women surveyed in the Northern Ireland study [48], however, did have a ‘named midwife’ who provided all or most of their care during pregnancy. 50% women saw just one or two midwives for their postnatal visits, the other 50% saw three or more midwives.

The NCT survey found that 71% of women appreciated seeing the same midwife for their care throughout the post-birth period [1]. In a Scottish survey [46], women reported how important it is to have the opportunity to develop relationships over time with professionals caring for them, both midwives and medical staff. The survey highlighted that women who received continuity of post-birth care reported excellent care from staff who listened and were sensitive to their needs. Unfortunately, the survey highlighted only 50% of women received continuity in their post-birth care. Women who did not receive continuity reported feelings of frustration and stress caused by inconsistent advice and support leading to dissatisfaction with recovery after birth. The survey highlights the importance of developing relationships over time and that these relationships that were highly valued by women during post-birth care.

Despite the desirability of relational continuity, lack of post-birth continuity is a recurrent theme across regions. Redshaw and Henderson found 40% of women in England had not met any of the midwives who provided post-birth care in the community and 33% saw three or more different midwives [12]. NPEU also reiterated the need in Northern Ireland for women to have individualised care delivered by 1–2 midwives [48]. Likewise, the RCM found only 4% of women had continuity of care across the childbirth year (antenatal, intrapartum and post-birth) from the same midwife. Although the RCM found 27% of the women had some care from the same midwife antenatally and postnatally only 50% had met a known midwife during the post-birth period [2]. The Support Overdue [44] report highlights how current post-birth care continues to be less than optimal and “dangerously fragmented” and needs to be redesigned with personalised relational continuity central to care as proposed by current UK policy.

### Trust and compassion

Building and sustaining trusting relationships over time appears to invoke a feeling of calmness and reduce stress. The RCM highlights how relational continuity facilitates trusting relationships between women and midwives and helps strengthen emotional, psychological, social and physical care [2]. The Care Quality Commission found a significant difference when continuity of care was analysed for the theme of ‘compassion’ with women experiencing less compassionate care when there was no consistent midwife during both antenatal and post-birth care [4]. They also reiterated that building relationships of mutual trust with health care professionals was essential for wellbeing and satisfaction and was particularly important during the post-birth period because this part of the childbirth continuum can be a particularly emotional stage in which established relationships are beneficial in transitioning to parenthood. It is evident that relational continuity enables personalised care planning helping informational continuity to occur over time in a trusting, context specific and sensitive way. The Department of Health highlighted the significance of ensuring continuity of support through the entire maternity experience via partnership with a health care professional that works towards provision of individualised, flexible and women-focused approaches [36]. The Scottish Government expects every woman to receive care from a primary midwife who provides most maternity care – antenatal, intrapartum and postnatal to enable women to develop trusting relationships with their midwife [29]. Evidence and policy clearly indicate that relational continuity enables a relationship of mutual trust that respects and supports woman’s decision-making.

### Supports decision making

NICE recommended that all planning of care should be personalised and documented to improve continuity of care and support decision-making [7]. Repeatedly, evidence and policy point to the significance of women needing care that is joined-up between health care professionals, hospital, and community. Relational continuity is recognised as supporting personalised care which maximises the opportunity for consistent advice to help women adapt to parenthood [4, 29, 33, 37, 46, 47]. The Scottish Government reiterates the importance of relational continuity as a way of facilitating individualised post-birth care that enables better informational and emotional support [46]. This need for personalised care is continually highlighted in evidence globally. In Belgium, Helsloot et al. emphasised the importance of continuity of care for meeting individual needs in a flexible way and improves acceptability [39]. Similarly, promotion of relational continuity across the maternity care pathway to improve post-natal care planning mirrors Australian policy [34].

### Supports post-birth care planning

Despite the vast evidence for relational continuity, conferring benefit, there remains a paucity of how this directly influences post-birth planning. It is apparent that relational continuity throughout the childbirth year is best enabled when a midwife or small team of midwives carry a caseload and responsibility for a group of women including post-birth care despite place of birth [33]. This Scottish policy document clearly indicates its wish for all women across Scotland to have a named midwife for a minimum of 10 days postnatally yet does not explicitly indicate that the same midwife provides care across the antenatal and post-birth period, or indeed the intrapartum. The Welsh Government in their survey concentrated on continuity of care yet again failed to detail how this would influence (or not) post-birth planning and care focussing mainly on antenatal and intrapartum with an emphasis on dignity and respecting women’s choices [49]. Whilst the importance of providing ‘personalised care plans’ is recognised by the NHS England [47] it only appears to extend to the antenatal period and birth, with no specific mention of extending this to the post-birth period. The NHS revisited this in their pathways for maternity care and once more reiterated the importance of maintaining continuity of care/ carer both antenatally and postnatally [32]. The North-West London STP [40] emphasises the need for using a PBCP during antenatal care through face-to-face conversations with their midwives. An overarching aim of the STP project [40] was to improve post-birth care and continuity of care, however, the evaluation of the project explicitly emphasised personalised care without overt focus on continuity of care despite the project’s team’s initial aspirations to be aligned with the recommendations for relational continuity set out by Better Birth [37].

Childbirth is dynamic and often unpredictable, any planning needs to appreciate this, and relational continuity would appear vital in enabling this. The NHS resource pack for implementing Better Births, suggests that post-birth planning is considered before birth and continually revisited in the postnatal period [38]. Although Forster et al. shows the importance of flexible and individualised care and how planning care antenatally is crucial for post-birth care, they only focussed on early discharge planning from hospital and did not emphasise the potential impact of relational continuity on such planning [11].

Despite relational continuity not being foregrounded explicitly across all 21 included papers it is plausible that such ongoing planning within a relational continuity relationship with care providers is advantageous and provides better support and building of trust enabling postnatal individual decisions to be made in partnership – including an optimal time for discharge home for those that birth within institutions. What is key is that maternity care is organised to reflect current evidence privileging and valuing relational continuity to help enable personalised post-birth care to be safe and acceptable so that women and families can flourish.

### Feasibility and acceptability in practice

The feasibility and acceptability of implementing PBCP in practice, including potential challenges, has been discussed [2] or at least highlighted [39, 40] in various papers. Moreover, there are other discussions elsewhere about ‘feasibility’ in relation to providing continuity of care [29] and individualised post-birth care [11]. As previously highlighted, there are very few examples of PBCP or maternity care plans available in the public domain and research evidence further substantiates that these are not being widely used in practice. In one survey, 96% of the women stated that they had not been not involved in developing a PBCP as recommended by NICE [1]. In another, almost half of the participants could not recall discussing a PBCP before the birth of their baby and two-thirds had not discussed it afterwards [2]. Two principle explanations for this relate to a lack of available resources [2, 40] and a lack of professional awareness of the NICE and other policy guidelines around post-birth care planning [2].

Essentially, a shortage of midwives in the UK has created various pressures, including a lack of time to adequately discuss and plan for women’s individual post-birth care needs. Only a third of midwives in one survey stated that potentially they had time to discuss and review PBCP with women in their care [2]. Over two-thirds of practising midwives and one-fifth of student midwives in one survey were unaware of the NICE recommendations [2], demonstrating ‘ … that this information is not being readily disseminated to front line staff, that they do not have time to engage with it in any detail and the content does not appear to be a policy driver’ [2]. Addressing resource concerns and this lack of awareness is critical in increasing the creation, implementation and usage of PBCP in practice.

## Discussion

The findings of this scoping review have demonstrated minimum empirical evidence of PBCP. Conversely, there are several evaluation surveys and policy documents that indicate that women appreciate post-birth planning, consistent advice and desire continuity of support preferably with a care provider, in the main a midwife, who they have come to know over time. The data suggest that there is a need for a PBCP and that this has to personalised, context specific and delivered flexibly over time in a conversational way to reflect the dynamic nature of the childbirth year. The findings of this scoping review indicate that emergent evidence and current policy are increasingly consistent, however, practice implementation of PBCPs is consistently poor. Post-birth care continues to be treated as the poor relation in maternity care despite the nature of this critical time for women and families.

The findings clearly highlight the significance of relationships and we propose that implementing PBCP within a fragmented system not based on relational continuity is worthwhile but would have limited effect. The fundamental importance of relational continuity in post-birth care planning has resonated throughout this review. This is concerned with developing a relationship over time, rather than merely a rapport. A relationship better privileges a conversational approach to care planning and moves away from tick box questioning. The PBCP as a standalone tool is questioned and needs to be part of a wider maternity care planner. Our interpretation of the findings leads us to recommend implementing both a model of care that enables relational continuity and post-birth care planning. We contend that introduction of both these interventions together would not only improve satisfaction but influence bio-medical, psychological and social outcomes. However, this review is not about demonstrating causative pathways and there is a need for more research to explore this in further depth.

## Conclusions

Although there has been a shift in health policy reflecting research evidence there remains a gap between this and the reality in practice for many women. Despite a significant body of evidence and policy recommendations to support continuity of care and implementation of post-birth care planning there continues to be a slow uptake regionally and internationally. A positive post-birth experience for women and their families is important. Consistently feedback from women highlights a tendency towards dissatisfaction with post-birth care, not only within the UK context, but also in the U.S., Australia and Belgium where maternity services are based on different models of care. This review illustrates how PBCPs coupled with relational continuity delivered through continuity of carer models of midwifery care highlights an opportunity to improve post-birth experiences for women and families. Conjoining continuity of carer and PBCP ensures that introduction of any PBCP remains dynamic and personalised avoiding any tendency towards a formulaic tick box approach.

This review is a first attempt at consolidating the literature around post-birth care planning and provides a starting point to evaluate PBCB. The review highlights the women’s desire for face-to-face discussions on post-birth planning with a small group of known professionals over time. For this opportunity to become reality evidence and policy must be implemented in equitable ways.

### Strengths and limitations

This scoping review followed a robust process through identification of a clear aim, to database searching, study selection and data extraction, following the model prescribed by Arksey and O’Malley [31]. The research team worked independently on study selection and came together collaboratively on all aspects of the review. The challenges were identifying and interpreting the terms and language used to describe PBCP in the literature and comprehending a variety of methodologies and article types. In addition, no study specifically addressed the questions posed for this review and multiple reviews of each article was required prior to inclusion. As a scoping review critical appraisal of the articles was not done. Indeed, “… the scoping study does not seek to assess quality of evidence and consequently cannot determine whether particular studies provide robust or generalizable findings” [31].

Although this may-be viewed a limitation of the review, we have provided a robust and in-depth overview of the domain and provided insightful synthesised findings that contribute to the body of knowledge concerning PBCPs. This review does not claim generalisability to all contexts. However, the principal themes identified in this review are transferable to similar contexts providing a much-needed dialogue around PBCP. The outcome of the review has revealed that PBCPs are worth exploring and that together with relational continuity of care models are helpful, desired and acceptable.

### Future research

This review has identified several questions requiring further research:

- Does PBCP improve outcomes?
- How does use of PBCP within a fragmented (non-continuity of carer model of care) correlate with women’s satisfaction and bio-medical, emotional and social outcomes?
- How does use of PBCP within a relational continuity care model correlate with women’s satisfaction and bio-medical, emotional and social outcomes?
- Where, when and how should a PBCP be used?
- What is the optimal and most useful informational content and style/format of a PBCP?
- This review was mainly focussed on evidence and policy that came from the UK, with some insights from the US, Australia and Belgium. What is now required is further PBCP related empirical studies that explicitly focus on cultural and socio-economic diversity to establish if the same themes identified in this review would be repeated across different geo-political regions, and minority and socioeconomic groups.
